# Is mouse embryonic stem cell technology obsolete?

**DOI:** 10.1186/s13059-015-0673-6

**Published:** 2015-05-27

**Authors:** William C Skarnes

**Affiliations:** Stem Cell Engineering, Wellcome Trust Sanger Institute, Wellcome Trust Genome Campus, Hinxton, Cambridge, CB10 1SA UK

## Abstract

Injection of recombinant Cas9 protein and synthetic guide RNAs into mouse zygotes has been shown to facilitate gene disruption and knock-ins using the CRISPR system. These technologies may soon displace genetic modification using embryonic stem cells.

## Genetic engineering of the mouse

Since the discovery of mouse embryonic stem (ES) cells more than 25 years ago, researchers have exploited their properties to engineer increasingly sophisticated genetic alterations with nucleotide precision. Mouse ES cells are highly amenable to genetic modification because of their rapid growth rate, ease of DNA transfection, and clonability. High rates of homologous recombination between an exogenous donor vector and the endogenous genome are routinely achieved in mouse ES cells; they are highest when linear isogenic constructs are used, with long homology arms and cassettes for positive and negative selection. Over the years, incremental improvements in gene-targeting vector design have made it possible to engineer mice carrying virtually any desired genetic modification, from a single base-pair change to the replacement of megabase-sized regions of the genome. Mouse ES cell technology embodies the most sophisticated forms of genome engineering practiced yet, culminating, for example, in genome-scale conditional knockout resources for the functional annotation of mammalian genes [1] and the humanization of the mouse immunoglobulin genes [2]. But the pre-eminence of mouse ES cells is now being challenged by technology involving clustered regularly interspaced short palindromic repeats (CRISPR) and the associated endonuclease Cas9 that enables genetic engineering of mouse embryos directly [3, 4]. In a recent paper in *Genome Biology*, Kohichi Tanaka and colleagues [5] describe an improved CRISPR-Cas method for efficient editing of mouse embryos by homologous recombination.

One significant drawback of mouse ES cell technology is the time and effort required to produce genetically modified mice from ES cells. At least 6 months is required to produce and breed ES-cell-derived chimeras from injections of modified ES cells in to pre-implantation embryos, with no assurance that the genetic modification will be transmitted to their offspring. Not all ES cell lines are robust, especially those derived from inbred mice other than the 129 strain, and often multiple independent targeted clones must be injected to produce mice. Genome instability of cultured ES cells is one reason for failure of germline transmission, overtly manifested by the loss or gain of whole chromosomes in culture. Regional copy number changes are also common in cultured ES cells [6] and are of particular concern if small sequence gains or losses are transmitted to offspring. In practice, germline transmission of more than one independent ES cell clone is required to establish a causal link between a genetic alteration and the phenotype observed in mice. Despite these difficulties, more than 25,000 genetically modified mouse strains have so far been produced from ES cells, including knockouts of about half the protein coding genes in mice.

## Nuclease-assisted targeting

Soon after gene targeting was established in mouse ES cells, researchers began to ask if homologous recombination was possible in one-cell embryos. Only one group managed to produce a single recombinant mouse from pronuclear injections of more than 10,000 embryos [7]. Clearly, the rate of homologous recombination in fertilized eggs was too low to provide a viable alternative to ES cell technology. Around the same time, experiments in mouse ES cells by Maria Jasin and colleagues [8] showed that gene targeting with a homologous donor plasmid is increased at least 50-fold by inducing a double-strand break in the target locus with the meganuclease I-SceI. Double-strand breaks not repaired by homologous recombination were often associated with small insertions or deletions characteristic of error-prone non-homologous end joining (NHEJ). These pioneering experiments would lay the foundation for genome editing of mouse embryos following the advent of programmable site-specific nucleases, initially using zinc finger nucleases [9] and more recently CRISPR-Cas9 nucleases [3, 4].

Researchers have embraced CRISPR-Cas9 to generate rudimentary alleles in mouse zygotes and other model species. Simple insertions/deletions induced by NHEJ or the incorporation of single strand oligonucleotides by homology-directed repair are now routinely carried out by transgenic facilities. However, more complex alleles that require homologous recombination with a donor plasmid [4, 9] are more difficult to produce by zygote injection. Typically, only a few percent of injected embryos co-injected with a nuclease and a donor plasmid carry the desired targeted modification. Because the founder animals are often mosaic, transmission of the targeted allele to the next generation is not guaranteed. Thus, further improvement of Cas9-assisted targeting in mouse zygotes is required to rival the broad range of genetic modifications possible in mouse ES cells.

## Cloning-free CRISPR-Cas9

The standard method for co-delivery of Cas9 endonuclease and a donor plasmid is to inject a mixture of the donor plasmid, Cas9 mRNA, and guide RNA into the cytoplasm and/or pronucleus of the fertilized egg [4]. Alternatively, pronuclear injection of recombinant Cas9 protein in complex with guide RNA also proved to be very effective in generating simple knockouts in mouse and zebrafish [10]. Based on delivery of Cas9 ribonucleoprotein (RNP), Aida *et al.* [5] have now developed a highly efficient method for the production of knock-in alleles in mice. Injections of Cas9 RNP and a donor plasmid encoding green fluorescent protein (GFP) into the pronucleus of one-cell embryos resulted in correct homologous recombination in 5 of 11 founder animals. In contrast, only one of eight founders carried the correctly targeted allele from injections of standard Cas9 mRNA, guide RNA and donor plasmid. Interestingly, chemically synthesized dual guide RNAs (CRISPR RNA and trans-activating CRISPR RNA) seem to be more effective than *in vitro* transcribed single guide RNA, although no difference in Cas9 endonuclease activity was observed in an *in vitro* digestion assay. This finding suggests the assembly of active Cas9 ribonucleoprotein complexes in the embryo is not optimal using *in vitro* transcribed single guide RNA and merits further investigation.

Several advantages of using Cas9 ribonucleoprotein are highlighted in the study by Aida *et al*. [5]. First, the observed increase in homologous recombination efficiency is probably due to the fact that Cas9 protein complexes are immediately active. When delivered to the nucleus with a donor plasmid, Cas9 ribonucleoprotein promotes homologous recombination at the target site without delay. Second, embryos are exposed to high Cas9 activity for a short period of time, reducing the likelihood of mosaicism and off-target damage. Indeed, no detectable damage at predicted off-target sites was observed in founder animals and, consistent with non-mosaic inheritance, 50 % of their offspring carried the targeted allele. From a practical standpoint, the use of synthetic guide RNAs is very convenient, eliminating the need to clone and purify *in vitro* transcribed single guide RNA. The high rate of homologous recombination observed in founder animals using recombinant Cas9 protein and synthetic guide RNAs [5] is very encouraging. More studies are now needed to determine if this method is generalizable to other loci and other vertebrate species.

The remarkable rise of nuclease-assisted genome editing signals a revolution in genetic engineering that is applicable to any model species or cell. The CRISPR-Cas system, in particular, is simple to use and the reagents for modifying embryos and cells can now be purchased from commercial suppliers. We can expect continued improvements in the quality of these reagents and methods for their delivery. Lingering concerns about the specificity of Cas9 endonuclease are falling away with each report that documents little or no off-target damage in founder animals. In comparison with the genome instability observed in cultured ES cells (an issue never satisfactorily resolved with respect to ES-cell-derived models), CRISPR-Cas technology is relatively safe. The study by Aida *et al*. [5] shows that Cas9-assisted targeting is very efficient in mouse embryos and will enable the engineering of complex alleles. Thus, the end of mouse ES cell technology now seems inevitable.

## Abbreviations

CRISPR: clustered regularly interspaced short palindromic repeats
ES: embryonic stem
NHEJ: non-homologous end joining
RNP: ribonucleoprotein
